# Confectioners’ Colors

**Published:** 1890-03

**Authors:** 


### CONFECTIONERS' COLORS.

The confectioner who would sell his wares must not only gratify the palate, but please the eye. It is to meet this latter requirement that he colors his confections, and not to satisfy his own artistic proclivities.

As to the beautiful yellow of some of his handiwork, no confectioner will admit the soft impeachment that he uses anything but yolks of eggs, but that in some cases, at least, the *Gallus bauckwa, vardomesticus*, has never had anything to do with a yellow coloration is fairly assumable. In such cases turmeric is used, and is not only innocuous, but healthful, and in India is extensively employed in cookery, not only for its color, but also for its tonic and stimulant properties. A druggist in certain neighborhoods might find a ready sale for a concentrated decoction of it.

Green is generally a color looked upon with suspicion, suggesting thoughts of arsenic and the undertaker. Spinach is the thing now most extensively used in the trade, sap green having been ruled out of court. Druggists might prepare, and profitably sell, a green color for confectionery by beating one pound of spinach in a stone mortar, and afterwards rubbing it through a fine sieve. One pound of fine sugar is then to be incorporated with it. The quantities to be used for coloring will depend entirely on the shade of green required.

A chocolate color may be produced by essence of coffee, and burnt onions are the best browning for soups and gravies.

The coloring power of saffron is known to everybody, but the flavor it also imparts is not often required.—*Druggist.*

